# Genetic Similarity of *Avena sativa* L. Varieties as an Example of a Narrow Genetic Pool of Contemporary Cereal Species

**DOI:** 10.3390/plants10071424

**Published:** 2021-07-12

**Authors:** Magdalena Cieplak, Sylwia Okoń, Krystyna Werwińska

**Affiliations:** 1 Institute of Plant Genetics, Breeding and Biotechnology, University of Life Sciences, 20-950 Lublin, Poland; magdalena.cieplak@up.lublin.pl; 2 PBAI Group, Plant Breeding Strzelce Ltd., Główna 20, 99-307 Strzelce, Poland; k_werwinska@hr-strzelce.pl

**Keywords:** oat, genetic similarity, ISSR, SCoT, UPGMA, PCoA

## Abstract

The assessment of the genetic diversity of cultivated varieties is a very important element of breeding programs. This allows the determination of the level of genetic differentiation of cultivated varieties, their genetic distinctiveness, and is also of great importance in the selection of parental components for crossbreeding. The aim of the present study was to determine the level of genetic diversity of oat varieties currently grown in Central Europe based on two marker systems: ISSR and SCoT. The research conducted showed that both these types of markers were suitable for conducting analyses relating to the assessment of genetic diversity. The calculated coefficients showed that the analyzed cultivars were characterized by a high genetic similarity. However, the UPGMA and PCoA analyses clearly indicated the distinctiveness of the breeding programs conducted in Central European countries. The high genetic similarity of the analyzed forms allow us to conclude that it is necessary to expand the genetic pool of oat varieties. Numerous studies show that landraces may be the donor of genetic variation.

## 1. Introduction

The assessment of genetic diversity plays an important role in the characterization of breeding lines, cultivars, or species and is the basis for the selection of appropriate parental forms in the development of crossing. In the 20th century, more diverse and less productive landraces were increasingly replaced by modern elite cultivars. These cultivars were usually derived from crosses found among genetically related modern cultivars, which resulted in greater productivity but caused a significant reduction in genetic diversity [1,2,3]. This problem affects many species, such as wheat, barley, and maize [4,5,6].

The common oat (*A. sativa* L.) is one of the six most widely cultivated cereal species in the world. The world’s largest producers of oat, according to the FAO, are Russia; Canada; the United States; Australia; and European countries such as Poland, Finland, and Spain (FAOSTAT 2020). As in other cereals, in oats the high genetic similarity has also become a considerable problem for breeders [7,8]. Crossbreeding within a small pool of parental forms has resulted in most of today’s breeding cultivars being almost homogeneous and closely related [9]. The consequence of this is a relatively narrow gene pool, resulting in a low phenotypic diversity [7]. A reduction in genetic diversity may adversely affect many important traits—for example, the resistance to new pests and pathogens, as well as adaptations to climate change and agricultural practices [7,10]. Because of this, the loss of genetic diversity has become an important problem not only in oat breeding but also in many other cereal species. Therefore, research focused on genetic diversity plays a key role in modern plant breeding.

Genetic diversity studies based on different tools, including DNA markers, provide important information both for genetic protection and for the effective breeding of new cultivars. DNA markers have numerous applications in plant molecular genetic studies and are commonly used for phylogenetic and taxonomic studies, or in determining the degree of similarity and assessing genetic distance, molecular mapping, and plant selection [11,12,13,14,15].

Among the different marker systems, inter-simple sequence repeats (ISSRs) are an efficient type of marker system that is multi-locus, dominant, reproducible, and highly polymorphic for genetic diversity studies. ISSR repeats are present mostly in the non-coding regions of chromosomes and specific stretches of non-active DNA sequences. The high occurrence of ISSR between normal coding genes and their presence within certain chromosomes as satellite bodies makes the ISSR unique and advantageous for use in DNA fingerprinting [16,17]. Another marker type suitable for fingerprinting analysis is start codon targeted (SCoT) markers. These are based on polymorphisms in the short, conserved region of plant genes surrounding the ATG translation initiation codon [18,19].

Each marker type has its advantages and disadvantages; thus, the combination of several systems can be helpful for a better understanding of genetic diversity in analyzed species. In this study, we used the ISSR and SCoT markers to visualize the level of genetic diversity of oat varieties currently grown in Central Europe.

## 2. Materials and Methods

### 2.1. Plant Material and DNA Extraction

Plant materials used in this study included 36 oat cultivars currently grown in Central Europe (Table 1). Genomic DNA was isolated according to the modified CTAB method [20] from 10-day old seedlings and brought to a concentration of 20 ng/μL.

### 2.2. Molecular Analyses Using ISSR and SCoT Primer

The ISSR amplification was carried out according to a modified method described by Zietkiewicz et al. [16]. DNA amplification was carried out in a volume of 10 μL; the reaction mixture consisted of 2 μL of PCR Master Mix (Novazym Polska s.c., Poznań, Poland), 0.4 μL of ISSR primer, 1 μL of sterile water, and 60 ng of genomic DNA. The reaction was performed on a Simple Applied Biosystems thermal cycler using the following thermal profile: pre-denaturing for 5 min at 94 °C; 45 cycles at: 94 °C for 30 s, 48–52 °C (depending on primers) for 30 s, and 72 °C for 1 min, with a final incubation time of 10 min at 72 °C. The SCoT amplification was carried out based on the methodology described by Collard and Mackill [19]. A reaction mixture of 10 μL consisted of: 2 μL of PCR Master Mix (Novazym), 0.4 μL of primer, 1 μL of sterile water, and 60 ng of genomic DNA. The reaction was carried out in a PCR Simple Applied Biosystems thermal cycler using the following thermal profile: pre-denaturing for 3 min at 94 °C; 35 cycles at: 94 °C for 1 min, 50 °C for 1 min, and 72 °C for 2 min, with a final incubation of 5 min at 72 °C. The ISSR and SCoT amplification products were separated on a 1.5% agarose gel stained with 0.01% EtBr in TBE buffer (89 mM Tris-borate, 2.5 mM EDTA) for 1.5 h at 120 V.

### 2.3. Data Analysis

The results obtained were presented as a binary matrix in Excel. The presence or absence of a band was treated as a single feature and was assigned a value of 1 or 0, respectively.

The polymorphic information content (*PIC*) was calculated by applying the simplified formula [21]:

$$PIC=2fi\left(1-fi\right),$$

where *fi* is the percentage of the *i* th amplified band present.

The level of polymorphism of the primer (polymorphic products/total products) and the relative frequency of polymorphic products (genetic resources where polymorphic products were present/total number of genetic resources) [22] were calculated. 

The resolving power (*Rp*) of the primers was calculated using the formula:

Rp=∑Ib,

where *Ib*–band informativeness was calculated for each band scored by the primer individually.

$$Ib=1- [2\left(0.5-p\right)],$$

where *p* is the proportion of occurrence of bands in the genotypes out of the total number of genotypes. The resolving power of the primers is a very useful parameter for the molecular diagnosis of any species from a mixed population [23].

The genetic similarity index (SI) between all pairs of test forms was estimated according to Dice [24]. Principal coordinate analysis (PCoA) and cluster analysis using the UPGMA (unweighted pair group method with arithmetic mean) were performed based on the Dice algorithm using Past 4.03 [25]. Clustering was verified by bootstrapping.

## 3. Results

Of the initially analyzed 30 ISSR (University of British Columbia–UBC) and 20 SCoT primers designed by Collard and Mackill [19], 8 and 5, respectively, produced polymorphic and repeatable fragments and were selected to determine the genetic similarity between the 36 oat cultivars. PCR reactions with the ISSR and SCoT primers resulted in 103 and 58 amplicons, respectively. For the ISSR, out of the total number of products, 54 were polymorphic, representing 52.43% of the diversity. The number of polymorphic DNA segments based on SCoT was 27 (46.56%). The frequency of polymorphic products ranged from 0.32 to 0.76 for the ISSR and from 0.29 to 0.61 for the SCoT.

The resolving power of the ISSR primers ranged from 0.49 to 9.81, while that of the SCoT primers was in the range of 0.49 to 16.0. The average PIC value for the amplification products obtained from the ISSR primers was 0.168, ranging from 0.10 to 0.22. The average PIC value for the amplification products obtained from the SCoT primers was 0.154 and ranged from 0.08 to 0.21 (Table 2 and Table 3).

Based on the results obtained, a genetic distance matrix of 36 oat cultivars was created according to the Dice formula.

For the ISSR, the similarity index values ranged from 0.814 to 0.949, with an average of 0.885. For the SCoT, the similarity index values ranged from 0.786 to 0.978, with an average of 0.891. Combined ISSR and SCoT results showed that the similarity index value ranged from 0.817 to 0.946, with an average of 0.887.

**Figure 1:**
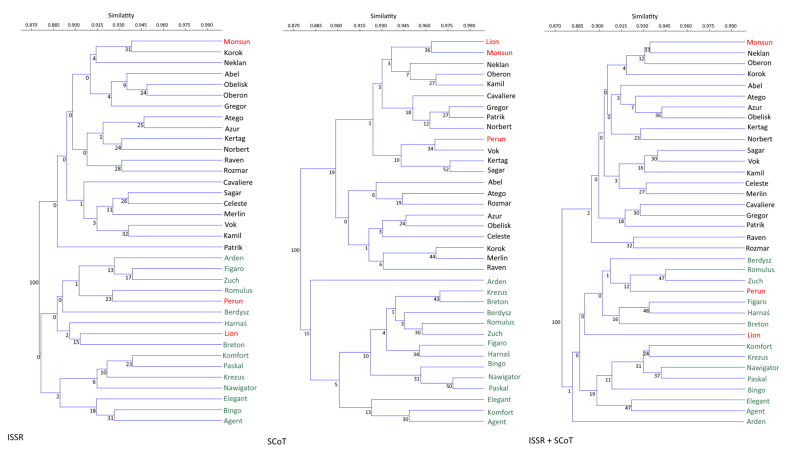
The UPGMA analysis of the 36 *A. sativa* L. cultivars. The origin of the analyzed varieties was marked with the following colors: green, Polish; black, Czech; red, German.

A genetic similarity matrix was applied for the cluster analysis using the UPGMA method. Dendrograms were constructed on the basis of the similarity indexes obtained with the ISSR and SCoT markers and based on a combination of both methods. In all dendrograms, the analyzed varieties were clustered into two main groups of clusters. The first cluster was dominated by the Polish varieties, and the second by the Czech varieties. The groupings of the German varieties in the first or second group were ambiguous, depending on the marker system (Figure 1).

**Figure 2:**
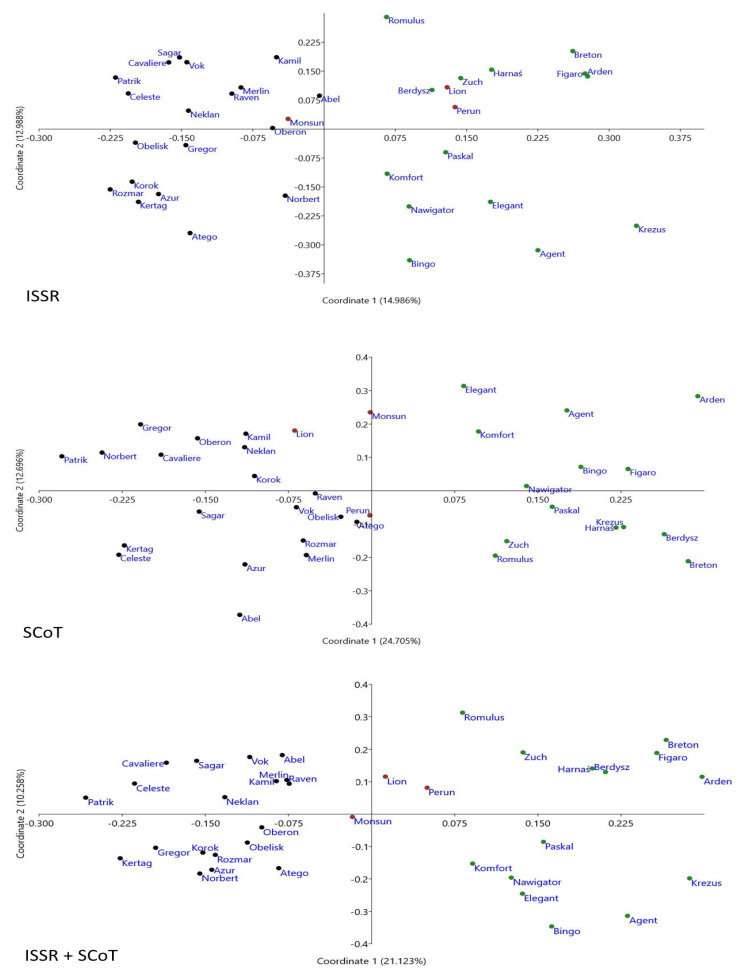
Biplot of the PCoA analysis of the 36 *A. sativa* L. cultivars. The origin of the analyzed varieties was marked with the following colors: green, Polish; black, Czech; red, German.

The PCoA analysis largely reflected the clustering of varieties with the UPGMA method. Across the three plots, the Polish and the Czech cultivars formed separate cluster groups. PCoA analyses also allowed the distinguishing of the German varieties, which can be seen in particular in the plot obtained based on the combined ISSR and SCoT methods (Figure 2). 

## 4. Discussion

The narrow genetic pool of cultivated varieties is a very serious problem for many cereals. Reduced genetic diversity can be seen particularly in studies comparing the genetic diversity of landraces and modern cultivars, indicating that it may be a result of carefully conducted breeding programs. Struss and Plieske [26] and Chen et al. [27] showed that barley landraces exhibited a higher level of polymorphism than cultivars. Similar results were obtained for wheat, where it was shown that landraces are more diverse at the genetic level than in modern cultivars [2,28,29]. Similar trends can also be observed in oat farming. Boczkowska and Onyśk [9] showed a much greater diversity in oat landraces from Poland compared to modern varieties. Montilla-Bascón et al. [7] analyzed and compared Spanish varieties. They put forward similar conclusions pointing to the greater genetic diversity in the landraces. In addition, Nersting et al. [30] showed that the Nordic landraces are characterized by a higher level of genetic diversity than the currently cultivated varieties. Fu et al. [31] also noticed a decrease in the number of alleles in modern Canadian varieties compared to landraces.

Data from the literature show that *A. sativa* varieties analyzed so far are characterized by a relatively narrow gene pool. This is especially shown for European varieties that are less diverse than those grown in North or South America [10,32].

Genetic diversity studies are based mainly on DNA markers. Oat cultivars have been the subject of many different studies aimed at determining their level of genetic variation. These studies were based on various marker systems such as RAPD [33], AFLP [32,33,34], ISSR [9,35], SSR [7,30,31], or DArT [36,37,38]. Different marker systems amplifying different regions of the genome can provide different information regarding the level of genetic diversity. However, in the case of oat varieties, the results obtained, regardless of the marker system used, indicate a high similarity between the genotypes cultivated today [9,33,35].

In the present study, the ISSR and SCoT markers were selected to evaluate the genetic similarity of chosen European oat varieties. Earlier studies have shown that ISSR markers are a good tool for assessing the level of genetic diversity of various cereal species [39,40,41,42,43,44,45,46,47,48,49,50,51,52], including oat [9,35]. The obtained frequencies of polymorphic bands in our own research confirmed the correctness of this choice for this marker system.

SCoT markers have been used numerous times to evaluate the genetic diversity of many cereals [46,48,53,54]. Until now, however, they have not been used in the genetic analysis of oat. The high polymorphism index of the markers used indicates that, similarly to ISSR, they can be used to evaluate the genetic pool of oats.

As in previous studies on the assessment of the genetic diversity of oat cultivars, the present study showed that the analyzed varieties were characterized by a low level of genetic diversity and a narrow genetic pool. The mean values of similarity obtained for the ISSR and SCoT markers were 0.885 and 0.891 and were similar to those obtained by Paczos-Grzeda [35], based on the ISSR (0.933) and RAPD (0.912) markers, and by Paczos-Grzeda [33], based on the AFLP markers (0.72) or based on the DArTseq markers (0.743) [38].

In the current research, based on the Dice genetic similarity index matrix, UPGMA cluster analysis and PCoA analysis were performed. The topology of the dendrograms was consistent with the arrangement of objects in the PCoA plots. Based on the analysis, it can be concluded that the grouping of varieties is consistent with their place of origin. Similar results were obtained by Paczos-Grzęda et al. [38], who showed that the grouping of Polish varieties depended on the place where they were bred. Newell et al. [37], He and Bjørnstad [55], and Tinker et al. [36] found that the clustering of individual lines was consistent with their origin. The conducted research also allowed for the identification of individual breeding programs. It was clearly visible that the Czech varieties underwent separate clustering from the Polish varieties. The German varieties were grouped together with the others, and this may be due to the fact that they were represented by only three genotypes. However, the PCoA analysis based on the combined results of the ISSR and SCoT clearly distinguished them as a separate group located between the Polish and Czech variants.

In summary, it can be concluded that the oat varieties currently grown in Central Europe are still characterized by a very low level of polymorphism. Both the ISSR and SCoT markers indicated a very narrow genetic pool; however, there are differences between the breeding programs carried out in individual countries. The conducted research indicates the need to expand the genetic variability of cultivated varieties. An excellent source may be, for example, local varieties, which are characterized by a very high level of genetic diversity compared to modern varieties [9,34].

## Figures and Tables

**Table 1:** 36 *A. sativa* L. cultivars currently cultivated in Central Europe.

Number	Variety	Country	Breeder
1	Agent	Poland	HR–Strzelce
2	Arden	Poland	DANKO
3	Berdysz	Poland	DANKO
4	Bingo	Poland	HR–Strzelce
5	Breton	Poland	MHR
6	Elegant	Poland	HR–Strzelce
7	Figaro	Poland	DANKO
8	Harnaś	Poland	MHR
9	Komfort	Poland	HR–Strzelce
10	Krezus	Poland	HR–Strzelce
11	Navigator	Poland	HR–Strzelce
12	Paskal	Poland	HR–Strzelce
13	Romulus	Poland	DANKO
14	Zuch	Poland	DANKO
15	Lion	Germany	Saaten–Union
16	Monsoon	Germany	Saaten–Union
17	Perun	Germany	Saaten–Union
18	Abel	Czech Republic	SELGEN
19	Atego	Czech Republic	SELGEN
20	Azur	Czech Republic	SELGEN
21	Cavaliere	Czech Republic	SELGEN
22	Gregor	Czech Republic	SELGEN
23	Kertag, New	Czech Republic	SELGEN
24	Korok	Czech Republic	SELGEN
25	Neclan	Czech Republic	SELGEN
26	Norbert	Czech Republic	SELGEN
27	Obelisk	Czech Republic	SELGEN
28	Oberon	Czech Republic	SELGEN
29	Raven	Czech Republic	SELGEN
30	Rosemary	Czech Republic	SELGEN
31	Sagar	Czech Republic	SELGEN
32	Vok	Czech Republic	SELGEN
33	Kamil	Czech Republic	SELGEN
34	Patrik	Czech Republic	SELGEN
35	Celeste	Czech Republic	SELGEN
36	Merlin	Czech Republic	SELGEN

**Table 2:** Characteristics of the ISSR primers used to assess the genetic similarity of the analyzed oat cultivars.

Primer	Sequence	Number of Products		Primer Diversity %	Frequency of Polymorphic Products	Resolving Power of the Primer (Rp)	Polymorphic Information Content (PIC)
		Total	Total				
SR 6	(GT)_8_ C	11	6	54.55	0.55	2.23	0.22
SR 17	(GA)_8_ YC	11	7	63.64	0.43	4.99	0.14
SR 32	(AG)_8_ YT	12	7	58.33	0.32	9.81	0.13
SR 39	(GA)_8_ GG	16	9	56.25	0.76	7.83	0.17
SR 42	(AG)_8_ YA	24	11	45.83	0.41	2.39	0.17
SR 50	(TC)_9_ C	10	6	60.00	0.42	2.64	0.22
SR 54	(CT)_9_ T	10	3	30.00	0.36	0.49	0.10
SR 59	(GATA)_6_ T	9	5	55.56	0.73	3.35	0.20
Total		103	54	52.43			
Average/primer		12.87	6.75				

**Table 3:** Characteristics of the SCoT primers used to assess the genetic similarity of the analyzed oat cultivars.

Primer	Sequence 5′->3′	Number of Products		Primer Diversity %	Frequency of Polymorphic Products	Resolving Power of the Primer (RP)	Polymorphic Information Content (PIC)
		Total	Polymorphic				
SCoT 3	CAA-CAA-TGG-CTA-CCA-CCG	13	6	46.15	0.61	16.0	0.21
SCoT 4	CAA-CAA-TGG-CTA-CCA-CCT	9	5	55.56	0.38	4.49	0.15
SCoT 12	ACG-ACA-TGG-CGA-CCA-ACG	9	4	44.44	0.61	3.14	0.12
SCoT 33	CCA-TGG-CTA-CCA-CCG-CCA	13	4	37.77	0.29	0.49	0.08
SCoT 34	ACC-ATG-GCT-ACC-ACC-GCA	14	8	57.14	0.40	0.67	0.21
Total		58	27	46.55			
Average/primer		11.6	5.4				

## Data Availability

Data are available upon request from the corresponding authors.
